# Descriptive analysis of esophageal cancer in Zambia using the cancer disease hospital database: young age, late stage at presentation

**DOI:** 10.11604/pamj.2021.39.12.23357

**Published:** 2021-05-04

**Authors:** Akwi Wasi Asombang, Nancy Kasongo, John Muyutu, Joao Filipe Goncalves Montiero, Nathaniel Chishinga, Jackson Chipaila, Lewis Banda

**Affiliations:** 1 Division of Gastroenterology, Hepatology, Warren Alpert Medical School of Brown University, Rhode Island 02903, United States,; 2 School of Medicine, Copperbelt University, Ndola, Zambia,; 3 Department of Medicine, Brown University, Rhode Island 02903, United States,; 4 Department of Surgery, University Teaching Hospital - Adult Hospital, Lusaka, Zambia,; 5 Hematology, Oncology, Cancer Disease Hospital, Lusaka, Zambia

**Keywords:** Zambia, Africa, esophageal cancer

## Abstract

**Introduction:**

published data on oesophageal cancer (EC) in Zambia is limited and our study is the only study in Zambia evaluating the demographics and clinicopathologic features of patients presenting with EC at time of diagnosis.

**Methods:**

a retrospective analysis of data from Cancer Diseases Hospital (CDH) database was conducted on EC patients diagnosed between 2007 and December 2018. Medical records of EC patients were manually retrieved and reviewed using medical record numbers identified from the CDH database. Demographics, clinicopathologic features and modes of treatment were extracted. A coding sheet was created a priori, and data analysed in SAS version 9.3.

**Results:**

two hundred and seventy eight (278) complete EC medical records were included in the analysis, 183 (66%) were males, mean age was 55 years (range 21-89). One hundred and fifty six (156) (56%) resided in Lusaka, the location of CDH. The age-standardized incidence for EC was 5.5 per 100,000 people (95% CI, 4.3-6.6). The commonest symptom was dysphagia (83%), 97% were diagnosed endoscopically, squamous cell carcinoma and adenocarcinoma accounted for 90% and 8.3% respectively, 65% received treatment. One hundred and twenty four (124) medical records had missing cancer staging. Of 154 medical records with complete cancer staging, 98 (35%) were diagnosed at stage 4 of which 33% were between 40 and 49 years.

**Conclusion:**

the age-standardized incidence for EC is high at CDH. Patients with EC are predominantly male, reside in Lusaka and present with late stage EC at time of diagnosis; mostly between the ages of 40-49 years. Robust prospective research and improved data recording is needed.

## Introduction

Africa is currently the epicentre of both communicable and non-communicable diseases. The contribution of esophageal cancer (EC) on cancer care in Africa is high and Zambia is no exception. The incidence and mortality of EC in Africa continues to rise. Greater than eighty percent of EC deaths globally occur in developing regions like Africa [1,2]. The epidemiology of EC in Africa is marked by significant variations in geographic distribution, age and sex. For instance, a recent systematic review and meta-analysis of esophageal cancer in Africa described incidence rates as low as 0.6 per 100 000 in Algeria to as high as 30 per 100 000 in Malawi [3]. Though literature notes that the incidence of EC in Africa appears to increase significantly after forty years other studies have demonstrated a notable occurrence of the disease in individuals below the age of forty [1,2]. The hall mark of EC sex distribution in most of Africa is male predominated though a deviation is seen in North Africa were the converse is true [2-4].

The clinical presentation of EC is often asymptomatic thus a diagnosis is usually reached when the disease has advanced leading to poor prognosis [5,6]. However, the lack of structured systems for EC screening and prevention poses a challenge for patients to access timely care. A study that assessed risk factors for squamous cell EC in Zambia found that on average patients presented to a health centre seventeen weeks after onset of disease symptoms [7]. Another study in South Africa that assessed predictors of late presentation found that the presence of advanced dysphagia, malnutrition (Body mass index (BMI) <18.5 kg/m^2^) and poor tumour differentiation were associated with late presentation to a health centre [8]. Findings of the above two studies indicate an urgent need for increased awareness on EC in developing countries. Some of the prominent risk factors for EC that have been highlighted across various literature include tobacco smoking and chewing, alcohol consumption, cooking with charcoal or firewood and HIV in individuals below the age of sixty [3,7,9]. The common histological type of EC in most low-income countries is squamous cell carcinoma while adenocarcinoma represents only a small portion, less than 5% in most studies in Africa [1,6,7]. The treatment options for EC range from endoscopic pulsion intubation, stenting, chemotherapy, radiotherapy and esophagectomy. Of these the most favourable outcomes stem from esophagectomy and chemo-radiotherapy which showed better survival [3]. The aim of our study was to describe the demographics and clinicopathologic features of patients presenting with EC at time of diagnosis at the only cancer hospital in Zambia.

## Methods

**Study design and location:** a retrospective analysis of data from the Cancer Diseases Hospital (CDH) database was conducted on patients diagnosed with EC from time of CDH inception in July 2007 to December 2018. The CDH is the only cancer treatment hospital in Zambia and is located in the capital city Lusaka. All cancer and cancer suspect patients nationwide are referred for management at the CDH. The CDH has a capacity of 262 beds; has one linear accelerator, two cobalt machines and two brachytherapy machines. The CDH also houses two computed tomography machines, one magnetic resonance imaging machine, two ultrasound machines, a digital mobile X-ray device and a fixed X-ray machine.

**Data collection:** upon diagnosis of a cancer the patient´s name, age, cancer type, residence and medical record number are entered into an electronic cancer data base at CDH. The database is based on Microsoft Access software. The following search terms were entered into the database to identify patients with EC “esophageal cancer” “Oesophageal cancer” “cancer of esophagus” “cancer of oesophagus”. A list of patients with EC was generated and medical records were manually retrieved using the medical record numbers and reviewed. The following were extracted: demographics (age at presentation, gender, residence), clinicopathologic features (symptoms at presentation, mode of diagnosis, tumour location, stage, histologic type), presence of risk factors (smoking, alcohol, HIV) and mode of treatment (chemotherapy, surgery, palliation). A coding sheet was created a priori and data analysed using SAS 9.3. Clinical staging utilized the National Comprehensive Cancer Network guidelines version 2 2018 staging for esophageal and esophago-gastric junction cancers.

## Results

**Figure 1:**
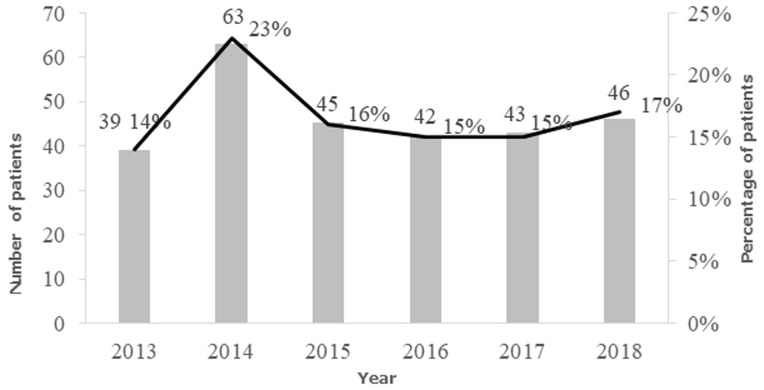
esophageal cancer (EC) trend at cancer diseases hospital, Lusaka 2013-2018, the black line represents the incidence of EC by year

**Figure 2:**
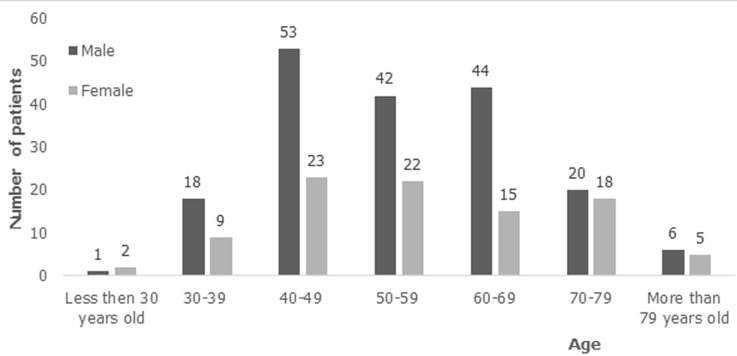
number of patients diagnosed with esophageal cancer by age range and gender

A total of 396 patients were identified in the CDH database for the period 2007 to 2018. Of these, 306 (77%) medical records were manually retrieved and 90 (23%) were missing. Of these 306 records, 26 (8%) were excluded because the patients had non-EC and 2 medical records had not information. Therefore, the final analyses included 278 medical records and these were for patients seen between 2013 and 2018. The year 2014 reported the highest number of EC cases (Figure 1). Of the 278 patients, 184 (66%) were males (Figure 1). The patients´ age ranged from 21 to 89 years with mean age 55 years. A majority of the EC cases were in the age group 40-49 years and nearly 90% of the cancer occurred from the fourth decade of life onwards (Figure 2). The age standardized incidence was 5.5 per 100 000 (95% CI, 4.3-6.6). Out of the 278 patients, over half (56%) were from Lusaka, the location of CDH and was followed by Copperbelt province 12% (north central region). The most common symptom of EC at time of diagnosis was dysphagia (88.8%), followed by weight loss (46%). Of the 278 medical records, 188 (68%) contained information on HIV status, of which 70% were HIV negative, 26% were HIV positive and 4% had unknown HIV status. Of the 278 medical records, 204 (73%) had information on alcohol intake and 205 (74%) on smoking. Of 204 records with information on alcohol, 64% had a history of alcohol intake, though 9% endorsed “stopping for years” and the rest were still taking as of the date of presentation at CDH (43.4%). For the smoking history, 107/205 (52.7%) medical charts retrieved had history smoking (Table 1). The body mass index (BMI) was calculated for 153 patients who had the necessary parameters documented for calculation. The range of the BMI was 8.1-39.8 with a mean of 18.0. Ninety-six patients (62.7%) were underweight (BMI<18.5).

**Table 1:** demographic and clinico-pathologic features of patients with esophageal cancer at CDH between 2007 and 2018

Characteristic	Number of EC patients (%)
**Province**	
Lusaka	156 (56.1)
Central	21 (7.6)
Copperbelt	33 (11.9)
Northern	3 (1.1)
Luapula	6 (2.2)
Eastern	26 (9.4)
North Western	2 (0.7)
Western	1 (0.4)
Southern	27 (9.7)
Outside Zambia	3 (1.1)
**Symptoms**	
Dysphagia	247 (88.8)
Weight loss	129 (46.4)
Odynophagia	34 (12.2)
Vomiting	23 (8.2)
Chest pain	21 (7.6)
Cough	19 (6.8)
Abdominal pain	17 (6.1)
Body weakness	6 (2.1)
Swollen neck	2 (0.7)
Constipation	1 (0.4)
Hematemesis	1 (0.4)
Excessive salivation	1 (0.4)
**Tumour location**	
Upper	41 (14.7)
Middle	105 (37.8)
Lower	88 (31.7)
**HIV status**	
Positive	49 (26.1)
Negative	131 (69.9)
Unknown	8 (4.3)
**Alcohol**	
Current	107 (55.5)
Quit	19 (9.3)
No history of use	78 (38.2)
**Smoking**	
Current	89 (43.4)
Quit	18 (9.3)
No history of use	98 (47.8)

EC: esophageal cancer; CDH: Cancer Diseases Hospital; HIV: Human Immunodeficiency Virus

**Figure 3:**
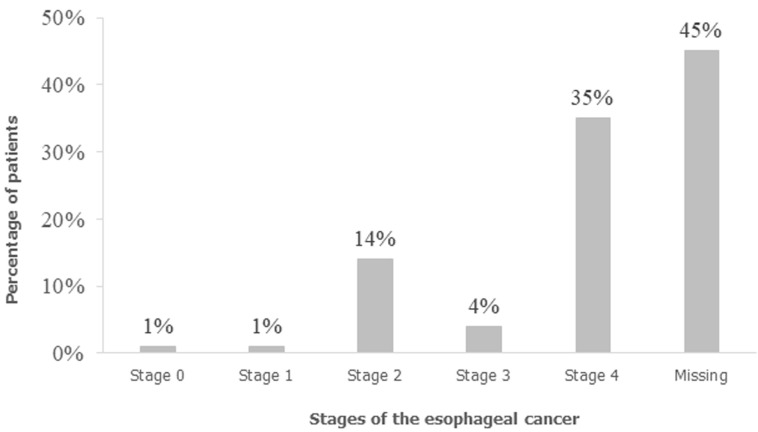
esophageal cancer clinical staging

Mode of diagnosis was by endoscopy in 97% of patients, while the mode of diagnosis was not indicated in 3% of the records. The cancer was primarily located in the mid esophagus in 39% of charts. On histopathology, squamous cell carcinoma accounted for 90% and followed by adenocarcinoma (8.3%) other histologies described were undifferentiated carcinoma 1% and carcinoma in situ 0.7%. CT scan was used for staging in 149/278 (53.6%), an abdominal ultrasound and chest X-ray used in 5/278, an imaging modality was not used in 124/278 (45%) for staging therefore one hundred twenty-four charts had missing staging data. Of the 154/278 patients with staging data, 98 (35%) were diagnosed at stage 4 (Figure 3) and of these 33% were between 40 and 49 years. One hundred and eighty-three (66%) had received treatment, while the form of treatment was not recorded in 95 (34%). Among those that received treatment 88/183 (48%) had chemo-radiotherapy, radiotherapy accounted for 79/183 (43%), chemotherapy alone 11/183 (6%) and palliation in 5/183 (2.7%).

## Discussion

This study describes clinicopathologic and demographic features of patients with esophageal cancer at the country´s only cancer treatment hospital. Data demonstrates that patients at CDH with EC are primarily younger than 50 years, and among those that present late, most are males. The male predominance of EC in our study is consistent with findings from other Southern African studies [1,5,6]; though other authors argue that this sex predominance could be a reflection of inequalities that exist in access to healthcare [4]. This picture could be supported by the prominence of high-risk habits for EC such as smoking and alcohol consumption in males in Sub-Saharan Africa [4,10]. Thirty-five percent of patients presented with advanced disease at diagnosis underscoring the need for screening for the cancer. The natural progression of EC, unlike other cancers, has been shown to take a long course before progressing to advanced disease, hence early diagnosis could lead to timely treatment and better outcomes [11].

The age-standardized incidence of EC at the CDH was 5.5 per 100,000. This is higher than incidences reported in West African and some North African countries with less than 1 per 100,000 reported for esophageal cancer [3]. Zambia is among countries with high EC incidence in Africa described as the “esophageal squamous cell cancer corridor” which extends from Sudan, East Africa through to South Africa with rates as high as 30 per 100,000 [4,6]. Over half of patients with EC at the CDH where from Lusaka, the city where the cancer hospital is located. This could be an indication of the existing difficulty in navigating care for EC for patients in other provinces and also a need for decentralization of cancer care through creation of smaller treatment hubs in the existing referral hospitals. This finding could also highlight a possible loop-hole in which many patients who are eligible for care from referral hospitals outside Lusaka are lost due to long distance to the treatment centre or existence of risks for the disease that make it more prominent in Lusaka.

Though 97% of EC in our study was diagnosed through endoscopy, 45% of the patients had no imaging studies to stage their disease indicating a burden of cost in care for the disease. Diagnosis and staging of cancers are particularly important in initiating appropriate treatment and follow up. As of 2018, the cost of an upper gastrointestinal endoscopy was approximately $100 US dollars ($), while CT scan $150 in most government hospitals in Zambia. This, in addition to other costs related to hospital care raises the overall cost of esophageal cancer treatment, while the daily income of majority of Zambians at risk of the disease lies below $1.9 as shown by the World Bank Group 2019 [12]. In view of this, our study supports the notion suggested by a study from Mozambique [2] that suggested the need to develop a holistic EC program to reduce disease incidence. The program would encompass all aspects of care from screening, awareness, diagnosis, staging, advocacy, training, treatment and research for EC. Among patients who received treatment, the dominant treatment modality for EC in our study was chemo-radiotherapy , possibly indicating the availability of both therapies in Zambia as opposed to other neighbouring countries were either radiotherapy or chemotherapy is more available [1,6]. The limitations include missing data due to the retrospective nature of our study. Particularly, data on surgical treatments modalities was missing including post-surgical outcomes. Since the incidence of EC is based on patients seen at the CDH, our findings cannot be generalized to incidence in the general population in Zambia. The strengths of this study include that the EC cases reported our study were histologically proven in 97% and staged by CT scan in over 50%, therefore represent an accurate demographic of patients attending the CDH. Further research maybe needed that determines the number of EC patients that fail to reach or access services at the CDH; the cost of treatment; and survival following treatment in Zambia.

## Conclusion

We found that a majority of patients with EC present with stage disease and most are males. A multidisciplinary program is needed to support nationwide awareness, screening, diagnosis and treatment of EC in Zambia.

### What is known about this topic

- The incidence of esophageal cancer has marked variations with high rates of incidence in East and Southern Africa;The incidence of esophageal cancer has marked variations with high rates of incidence in East and Southern Africa;
- The common histological type in developing countries is squamous cell carcinoma.

### What this study adds

- Our study contributes information that enables understanding the epidemiology of EC in an African population;
- Data demonstrates that patients at CDH with EC are primarily younger than 50 years, and among those that present late, most are males indicating a need for research to understand this trend;
- This study also provides information needed to raise awareness amongst health care providers and provide baseline data for future research studies on EC.
